# Regulating effect of dietary antioxidant quality on association between estimated glucose disposal rate and moderate to severe periodontitis

**DOI:** 10.3389/fnut.2025.1561497

**Published:** 2025-05-30

**Authors:** Ge Wang, Yujie Zhang, Fengli Li

**Affiliations:** ^1^Department of Conservative and Endodontic Dentistry, East Branch, Jinan Stomatological Hospital, Jinan, China; ^2^Department of Orthodontics, Shandanorthroad Branch, Jinan Stomatological Hospital, Jinan, China; ^3^Department of Maxillofacial Surgery, Shungeng Branch, Jinan Stomatological Hospital, Jinan, China

**Keywords:** eGDR, DAQS, periodontitis, NHANES database, regulating effect

## Abstract

**Aim:**

To investigate the associations of the estimated glucose disposal rate (eGDR) and the Dietary Antioxidant Quality Score (DAQS) with periodontitis, and assessed the potential regulating effect of DAQS on relationship between eGDR and periodontitis, to provide some references for management and control of periodontitis.

**Methods:**

Data of 9,588 individuals were extracted from the National Health and Nutrition Examination Survey (NHANES) database in 2009–2014 in this cross-sectional study. Associations of eGDR and DAQS with periodontitis were evaluated by multivariate logistic regression analysis, with odds ratio (OR) and 95% confidence interval (CI). The potential regulating effect of DAQS on association between eGDR and periodontitis was investigated using multiplicative interaction term, and analyzed in subgroups of age, gender, overweight and diabetes mellitus (DM). Additionally, the value of eGDR on periodontitis identification was compared with common insulin resistance (IR)-related indexes via the receiver operating characteristic (ROC) curve with area under the curve (AUC) and Delong test.

**Results:**

Among eligible participants, 3,111 had periodontitis stage I/II, and 6,477 had periodontitis stage III/IV. After adjusting for the selected covariates, an eGDR of <8.296 was associated with increased odds of moderate to severe periodontitis (OR = 1.28, 95%CI: 1.07–1.53), comparing to that of ≥8.296. An eGDR level of <8.296 was positively associated with increased odds of periodontitis, when individuals with a DAQS of <3 (OR = 1.67, 95%CI: 1.13–2.48), and however, this relationship was not significant among participants with DAQS of ≥3 (*p* = 0.051). The significant multiplicative interaction terms of eGDR and DAQS were observed in age ≥60 years (OR = 3.86, 95%CI: 1.95–7.62), female (OR = 1.68, 95%CI: 1.14–2.45), BMI ≥ 25 kg/m^2^ (OR = 1.53, 95%CI: 1.01–2.33), and non-DM (OR = 1.52, 95%CI: 1.06–2.18) subgroups. Furthermore, the ROC curve showed that eGDR has a relatively good value on periodontitis identification comparing to other IR-related indexes, with an AUC (95%CI) of 0.603 (0.585–0.620), and Delong test *p*-values of <0.001.

**Conclusion:**

A higher DAQS had a potential antagonistic effect on increased moderate to severe periodontitis risk associated with lower eGDR level. Clinicians could focus on eGDR level that may identify the potential risk of periodontitis, and improving DAQS levels may help the prevention and intervening measure in periodontitis.

## Introduction

In recent years, the prevalence of periodontitis has remained high all over the world, resulting in a huge burden of disease (1–3). Studies have reported that insulin resistance (IR) may be related to the disease and progression of periodontitis (4–7). The relevant mechanisms may include IR-related oxidative stress (OS), inflammation and their cross-talk change the oral immune microenvironment and saliva microbiome (8–10); and IR reduces the expression of vascular cell adhesion molecules, thus aggravating the inflammatory response of periodontal tissue by interfering with insulin-mediated Forkhead box O (FoxO) 1 activity (11). However, the complex detection methods of insulin limit the evaluation of IR, and thus the index that indirectly reflects IR has attracted the attention of researchers. Among them, the estimated glucose disposal rate (eGDR), is calculated by glycosylated hemoglobin (HbA1c), waist circumference, and hypertension, and a lower eGDR level has been shown to be strongly associated with diabetic complications (12–14) and non-diabetic cardiovascular disease (CVD) risk (14, 15). Moreover, the predictive value of eGDR for CVD mortality in patients with diabetic retinopathy and non-alcoholic fatty liver disease was higher than other IR indexes (16, 17). Nevertheless, until now, no studies have investigated the relationship between eGDR and periodontitis.

Antioxidant nutrients affect the development of inflammatory diseases associated with IR/metabolic abnormalities (such as CVD) through ameliorating OS and inflammation, and their deficiency may also exacerbate diabetes-related periodontitis (18, 19). However, there is a lack of research evidence on the effect of antioxidant nutrients on the risk of IR-related periodontitis.

The Dietary Antioxidant Quality Score (DAQS) is a composite index based on the intake of vitamin A (VA), vitamin E (VE), vitamin C (VC), magnesium (Mg), zinc (Zn) and selenium (Se), which comprehensively reflects the dietary antioxidant capacity (20). A higher DAQS were found to be associated with inflammation and components associated with metabolic syndrome (MetS) (21, 22), and to help improve the risk of death associated with high uric acid in patients with chronic kidney disease (CKD) (23). However, no studies have investigated the effect of DAQS on the risk of IR-related periodontitis.

Herein, this study aims to explore the association between eGDR and moderate to severe periodontitis, as well as the potential regulating effect of DAQS on this relationship, in order to provide some references for management and control of periodontitis.

## Methods

### Study design and subjects

In this cross-sectional study, data of participants were extracted from the National Health and Nutrition Examination Survey (NHANES) database in 2009–2014. The NHANES database is designed to sample the non-institutionalized population in the United States, using a complex, multistage stratified probability sample based on selected counties, segments, households, and individuals. Interviews in individuals’ homes conducted by the National Center for Health Statistics (NCHS) well-trained professionals, and extensive physical examinations were conducted at mobile exam centers (MECs).

A total of 10,714 adults with information on periodontal status in the NHANES database were initially included. The exclusion criterion was (1) missing information on eGDR calculation, including variables for measurement of HbA1c and waist circumference or hypertension diagnosis, (2) without information on intakes of VA, VE, VC, Mg, Zn or Se, and (3) edentulous. Finally, 9,588 of them were eligible. The NHANES survey was approved by the Institutional Review Board (IRB) of the NCHS of the United States Centers for Disease Control and Prevention (CDC). Since the study data were publicly available, according to relevant law and regulation, the ethical approval has been waived by the IRB of our hospital.

### Examination of eGDR

According to previous research (24), eGDR (mg/kg/min) was calculated using the following formula: eGDR = 21.158–0.09 ∗ waist circumference (cm) − 3.407 ∗ hypertension (defined as blood pressure higher than 140/90 mmHg on two occasions or using of antihypertensive medication, yes = 1/no = 0) − 0.551 ∗ HbA1c (%). In this study, eGDR was divided into two levels with the median value of 8.296 mg/kg/min.

### Calculation of DAQS

The DAQS was calculated based on six dietary antioxidant micronutrients, including VA, VC, VE, Zn, Mg, and Se. For the DAQS, each of the above six nutrients/minerals intake was compared to their respective daily recommended intake (RDI) for United States adults released by the Dietary Guidelines for Americans 2015–2020^1^. For each antioxidant vitamin/mineral, the DAQ scores of 0 and 1 were defined as intake <2/3 of the RDI and the intake ≥2/3 of the RDI, respectively, according to Rivas and colleagues’ method (25). The summed DAQS ranged from 0 (poor quality) to 6 (high quality). The DAQS was then classified into the three groups: 0–2 (low quality), 3–4 (medium quality), and 5–6 (high quality).

### Assessment of periodontitis

The periodontal assessment involved measuring clinical attachment loss (CAL) and probing pocket depth (PPD) at six predetermined sites on each tooth, following the full-mouth periodontal examination (FMPE) protocol and excluding the third molars. CAL refers to the distance between the cemento-enamel junction and the base of the sulcus, whereas PPD is the measurement from the free gingival margin to the base of the sulcus.

The classification of periodontitis was based on the definitions provided by EFP/AAP 2018 (26): the inter-dental CAL of two non-adjacent teeth, or the buccal or oral CAL, was ≥3 mm, with pocketing >3 mm. Severity staging was calculated accordingly: for each tooth, the CAL of the most severe site was recorded; a CAL of 1–2 mm was defined as Stage I, of 3–4 mm as Stage II and of ≥5 mm as Stages III-IV.

### Covariates selection

We also extracted data of variables that could be potential confounding factors from the database, including age, gender, race, educational level, marital status, poverty-to-income ratio (PIR), smoking, drinking, physical activity, diabetes mellitus (DM), hypertension, dyslipidemia, CVD, CKD, body mass index (BMI), decayed teeth, dental implants, frequency of using dental floss, white blood cell (WBC), total energy intake, and anti-diabetic drug use.

During the NHANES household interviews, participants who claimed to have smoked fewer than 100 cigarettes in their lives were divided into non-smoking group. The pattern of alcohol consumption was also captured by questionnaires (27). Overweight was defined according to the criteria of the WHO that body mass index of ≥25 kg/m^2^. Physical activity was converted into metabolic equivalent (MET), which was calculated according to the information collected by physical activity questionnaire in the NHANES: Energy expenditure (MET·min) = recommended MET × exercise time of corresponding activity (min), and categorized using the cut-off value of 450 MET·min/week. Patients with total cholesterol ≥200 mg/dL (5.2 mmol/L) or triglycerides ≥150 mg/dL (1.7 mmol/L) or low-density lipoprotein cholesterol ≥130 mg/dL (3.4 mmol/L) or high-density lipoprotein cholesterol ≤40 mg/dL (1.0 mmol/L) or self-reported hypercholesterolemia or receiving lipid-lowering therapy were identified as dyslipidemia (28). DM was defined according to a self-reported diagnosis, the use of oral hypoglycemic agents or insulin, HbAlc ≥6.5%, a plasma glucose level ≥200 mg/dL at 2 h after the oral glucose tolerance test, or a fasting glucose level ≥126 mg/dL. CVD was determined by multiple choice question in NHANES with the question: “Have you ever been told you had (congestive) heart failure, coronary heart disease, angina/angina pectoris, heart attack, or stroke.” Dietary intakes of total energy were calculated by “total nutrient intakes” and “total dietary supplements,” which collected via the NHANES 24-h dietary recalls.

Moreover, other indexes reflect IR were extracted, including the Homeostasis Model Assessment of Insulin Resistance (HOMA-IR), the Triglyceride/Glucose (TyG) Index, triglyceride (TG)/high-density lipoprotein cholesterol (HDL-C), and the Metabolic Score for IR (METS-IR) index (5, 29). HOMA-IR = fasting glucose (mmol/L) × fasting insulin (μU/mL)/22.5; TyG = ln [TG level (mg/dL) × FPG (mg/dL)/2]; METS-IR = ln [2 × fasting plasma glucose (mg/dL) + TG level (mg/dL)] × BMI (kg/m^2^)/ln [HDL-C level (mg/dL)].

### Statistical analysis

Data of continuous variables were expressed as mean and standard error (Mean ± SE), and *t*-test was used for comparison between Stage I/II periodontitis group and Stage III/IV periodontitis group. Data of categorized variables were described using frequency with constituent ratio [*N* (%)], and chi-square test (*χ*^2^) was used for the comparison. In the present study, dietary information used was collected during the first 24-h dietary recall, and according to the NHANES recommendation, the sample weights “dietary day one sample weight (WTDRD1)” should be used. The WTDRD1 weights were constructed by taking the MEC two-year cycle sample weights (WTMEC2YR), and further adjusting for (a) the additional non-response and (b) the differential allocation by day of the week for the dietary intake data collection.

Weighted logistic regression was utilized to screen covariates that significantly associated with periodontitis (with *p*-values of <0.05). Associations of eGDR and DAQS with periodontitis were explored using univariate and multivariate logistic regression, evaluated by odds ratio (OR) with 95% confidence interval (CI). The potential regulating effect of DAQS on relationship between eGDR and periodontitis was test using multiplicative interaction term, and then the effect of regulating variable (DAQS) on the association between the independent variable (eGDR) and the outcome variable (periodontitis) was quantified by different level groups of the regulating variable. Additionally, subgroups analysis of age, gender, BMI, and DM was performed. Model 1 was unadjusted model. Model 2 adjusted for demographic variables, including age, gender, race, education, marital status, PIR. Model 3 additionally adjusted for smoking, dyslipidemia, CVD, CKD, BMI, decayed teeth, frequency of using dental floss, WBC, and anti-diabetic drug use on the basis of the Model 2. Furthermore, we compared the identification values of different IR-related indexes on periodontitis, with the area under the curve (AUC) of receiver operating characteristic (ROC) curve, as well as the Delong test.

Statistical analyses were performed using R version 4.2.2 (Institute for Statistics and Mathematics, Vienna, Austria) and Python 3.9.0 (Python Software Foundation, Delaware, United States). Data of study variables with missing values were shown in the Supplementary Table S1, and they were interpolated via random forest interpolation method or categorized into “unknown” level. Sensitivity analysis on characteristics of participants before and after the interpolation of missing values was presented in the Supplementary Table S2.

## Results

### Characteristics of study subjects

**Figure 1:**
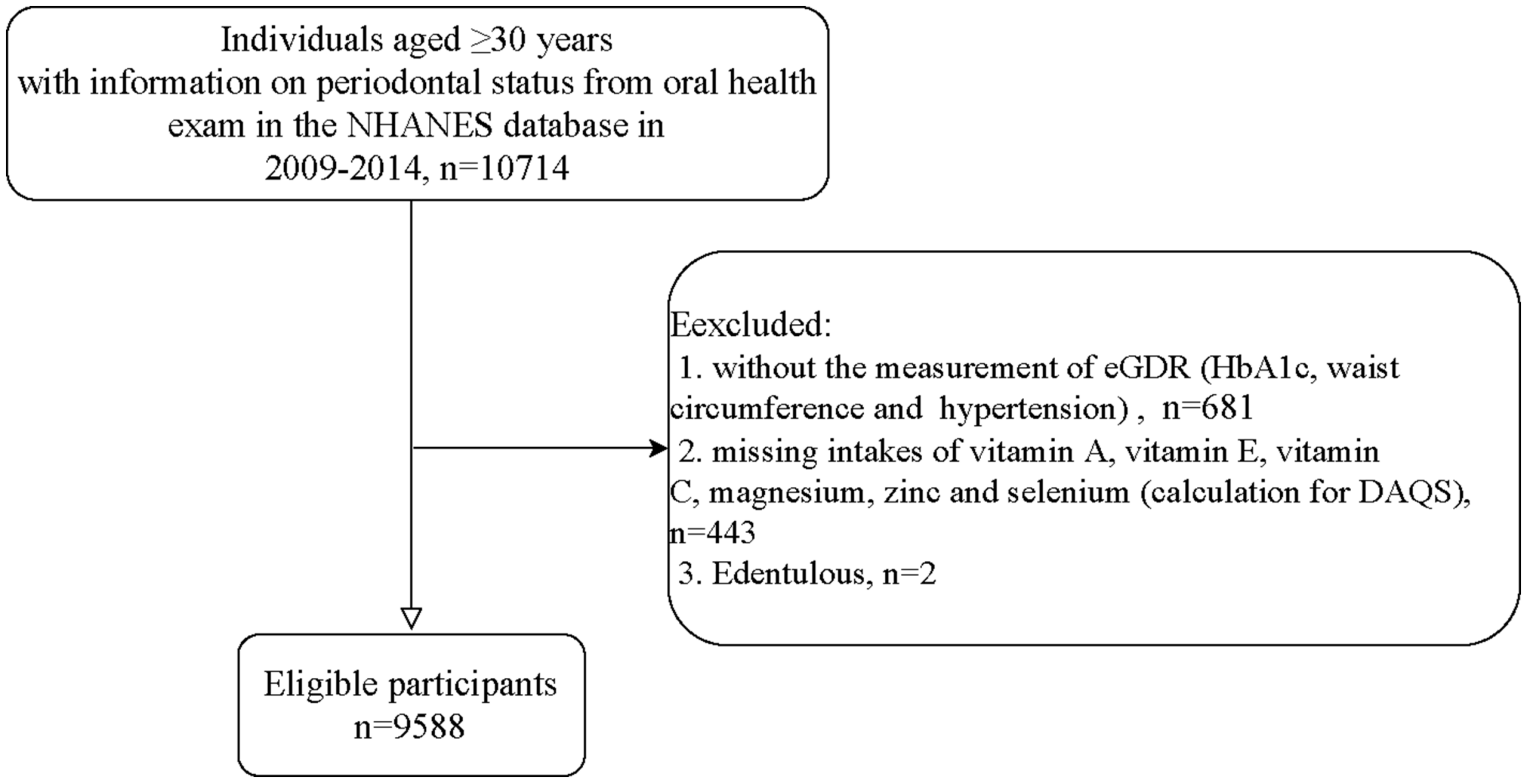
Flowchart of the participants screening.

The process of study participants screening is shown in the Figure 1. There are a total of 10,714 individuals aged ≥30 years old in the NHANES database in 2009–2014. Those who without information on variables for calculation of eGDR, including HbA1c, waist circumference or hypertension (*n* = 681), without information on intakes of VA, VE, VC, Mg, Zn or Se (*n* = 443), or edentulous (*n* = 2) were excluded. Finally, 9,588 of them were eligible.

According to the Table 1, study participants were divided into two groups according their periodontitis stages: stage I/II (*n* = 3,111) and stage III/IV (*n* = 6,477). The average age of total population was 50.85 years, and 4,755 (49.02%) were male. Mean values of DAQS in stage I/II periodontitis group and stage III/IV periodontitis group were, respectively, 4.19 and 3.87 (*p* < 0.001). Also, the average eGDR level was significantly higher in stage I/II periodontitis group than that in stage III/IV periodontitis group (8.34 vs. 7.29). Additionally, race, educational level, Marital status, PIR, smoking, physical activity, DM, hypertension, dyslipidemia, CVD, CKD, BMI, overweight, decayed teeth, frequency of using dental floss, WBC, and anti-diabetic drug were significantly different between these two groups (all *p* < 0.05). while alcohol consumption, dental implantation and total energy intake (kcal) were not (all *p* > 0.05).

**Table 1:** Characteristics of participants in different periodontitis groups.

Variables	Total (*n* = 9,588)	Stage I/II periodontitis (*n* = 3,111)	Stage III/IV periodontitis (*n* = 6,477)	Statistics	*P*
Age, years, mean (S.E)	50.85 (0.25)	46.56 (0.32)	53.57 (0.33)	*t* = −15.96	**<0.001**
Age, years, *n* (%)				*χ*^2^ = 95.317	**<0.001**
<60	6,540 (73.51)	2,531 (83.37)	4,009 (67.29)		
≥60	3,048 (26.49)	580 (16.63)	2,468 (32.71)		
Gender, *n* (%)				*χ*^2^ = 116.722	**<0.001**
Male	4,755 (49.02)	1,185 (39.96)	3,570 (54.74)		
Female	4,833 (50.98)	1926 (60.04)	2,907 (45.26)		
Race, *n* (%)				*χ*^2^ = 83.805	**<0.001**
Non-Hispanic White	4,261 (69.26)	1,662 (76.73)	2,599 (64.56)		
Non-Hispanic Black	1924 (10.36)	458 (6.92)	1,466 (12.54)		
Others	3,403 (20.37)	991 (16.36)	2,412 (22.91)		
Educational level, *n* (%)				*χ*^2^ = 210.194	**<0.001**
Below high school	2,182 (14.94)	417 (8.11)	1765 (19.25)		
High school	2074 (20.71)	535 (16.40)	1,539 (23.42)		
College and above	5,332 (64.36)	2,159 (75.49)	3,173 (57.33)		
Marital status, *n* (%)				*χ*^2^ = 75.509	**<0.001**
Married	5,638 (62.84)	1986 (69.79)	3,652 (58.44)		
Never married	1,092 (10.58)	378 (9.95)	714 (10.98)		
Others	2,858 (26.59)	747 (20.26)	2,111 (30.58)		
PIR, *n* (%)				*χ*^2^ = 167.802	**<0.001**
≤1.3	2,838 (19.12)	662 (12.07)	2,176 (23.57)		
1.3–3.5	3,450 (34.02)	1,001 (29.32)	2,449 (36.99)		
>3.5	3,300 (46.86)	1,448 (58.61)	1852 (39.44)		
Smoking, *n* (%)				*χ*^2^ = 79.881	**<0.001**
No	5,360 (55.63)	2046 (64.82)	3,314 (49.83)		
Yes	4,228 (44.37)	1,065 (35.18)	3,163 (50.17)		
Drinking, *n* (%)				*χ*^2^ = 2.700	0.100
No	2,532 (20.38)	780 (19.08)	1752 (21.20)		
Yes	7,056 (79.62)	2,331 (80.92)	4,725 (78.80)		
Physical activity, MET·min/week, *n* (%)				*χ*^2^ = 19.836	**<0.001**
<450	1,037 (10.56)	360 (10.40)	677 (10.66)		
≥450	6,205 (68.15)	2,102 (71.76)	4,103 (65.87)		
Unknown	2,346 (21.29)	649 (17.84)	1,697 (23.47)		
DM, *n* (%)				*χ^2^* = 53.348	**<0.001**
No	7,784 (85.88)	2,729 (90.19)	5,055 (83.16)		
Yes	1804 (14.12)	382 (9.81)	1,422 (16.84)		
Hypertension, *n* (%)				*χ*^2^ = 135.308	**<0.001**
No	5,422 (60.74)	2098 (70.17)	3,324 (54.79)		
Yes	4,166 (39.26)	1,013 (29.83)	3,153 (45.21)		
Dyslipidemia, *n* (%)				*χ*^2^ = 16.860	**<0.001**
No	2,380 (24.83)	925 (28.63)	1,455 (22.43)		
Yes	7,208 (75.17)	2,186 (71.37)	5,022 (77.57)		
CVD, *n* (%)				*χ*^2^ = 115.994	**<0.001**
No	8,801 (93.20)	2,972 (96.29)	5,829 (91.25)		
Yes	787 (6.80)	139 (3.71)	648 (8.75)		
CKD, *n* (%)				*χ*^2^ = 71.518	**<0.001**
No	8,359 (89.76)	2,856 (93.57)	5,503 (87.35)		
Yes	1,229 (10.24)	255 (6.43)	974 (12.65)		
BMI, kg/m^2^, mean (S.E)	29.11 (0.13)	28.52 (0.17)	29.49 (0.17)	*t* = −4.11	**<0.001**
Overweight, *n* (%)				*χ*^2^ = 16.277	**<0.001**
No	2,517 (27.10)	901 (30.27)	1,616 (25.11)		
Yes	7,071 (72.90)	2,210 (69.73)	4,861 (74.89)		
Decayed teeth, *n* (%)				*χ*^2^ = 278.014	**<0.001**
No	6,655 (75.25)	2,580 (87.90)	4,075 (67.27)		
Yes	2,933 (24.75)	531 (12.10)	2,402 (32.73)		
Dental implants, *n* (%)				*χ*^2^ = 0.025	0.873
No	9,330 (96.90)	3,015 (96.84)	6,315 (96.93)		
Yes	258 (3.10)	96 (3.16)	162 (3.07)		
Frequency of using dental floss, times/week, *n* (%)				*χ*^2^ = 13.011	**<0.001**
<3	4,561 (45.01)	1,343 (41.66)	3,218 (47.13)		
≥3	5,027 (54.99)	1768 (58.34)	3,259 (52.87)		
WBC, 1000 cells/uL, Mean (S.E)	7.12 (0.04)	6.89 (0.05)	7.26 (0.05)	*t* = −6.92	**<0.001**
Total energy intake, kcal, mean (S.E)	2150.48 (14.43)	2137.60 (17.90)	2158.61 (17.52)	*t* = −1.01	0.317
Anti-diabetic drug, *n* (%)				*χ*^2^ = 25.990	**<0.001**
No	8,472 (91.31)	2,883 (94.13)	5,589 (89.53)		
Yes	1,116 (8.69)	228 (5.87)	888 (10.47)		
DAQS score, mean (S.E)	4.00 (0.03)	4.19 (0.05)	3.87 (0.03)	*t* = 5.96	**<0.001**
DAQS, *n* (%)				*χ*^2^ = 30.643	**<0.001**
≥3	7,518 (81.58)	2,551 (85.61)	4,967 (79.03)		
<2	2070 (18.42)	560 (14.39)	1,510 (20.97)		
eGDR, mean (S.E)	7.69 (0.05)	8.34 (0.06)	7.29 (0.06)	*t* = 12.93	**<0.001**
eGDR levels, *n* (%)				*χ*^2^ = 143.133	**<0.001**
≥8.296	4,380 (50.01)	1761 (60.15)	2,619 (43.61)		
<8.296	5,208 (49.99)	1,350 (39.85)	3,858 (56.39)		

*t*, *t*-test; *χ*^2^, chi-square test.

The bold *p*-values represented statistically significant.

SE, standard; PIR, poverty-to-income ratio; DM, diabetes mellitus; CVD, cardiovascular disease; CKD, chronic kidney disease; BMI, body mass index; WBC, white blood cell; DAQS, the dietary antioxidant quality score; eGDR, estimated glucose disposal rate.

### Associations of eGDR and DAQS with periodontitis

Before investigating the associations of eGDR and DAQS with a periodontitis, covariates associated with periodontitis were screened (Supplementary Table S2). Then, after adjusting for the selected covariates, an eGDR of <8.296 was associated with increased odds of moderate to severe periodontitis (OR = 1.28, 95%CI: 1.07–1.53, *p* = 0.009), comparing to that of ≥8.296 (Table 2). However, no significant association between DAQS and periodontitis was observed when adjusted for all selected covariates (OR = 1.18, 95%CI: 0.99–1.40, *p* = 0.060).

**Table 2:** Associations of eGDR and DAQS with periodontitis.

Variables	Unadjusted model		Model 1		Model 2	
	OR (95% CI)	*P*	OR (95% CI)	*P*	OR (95% CI)	*P*
eGDR levels						
≥8.296	Ref		Ref		Ref	
<8.296	1.95 (1.72–2.21)	**<0.001**	1.42 (1.21–1.66)	**<0.001**	1.28 (1.07–1.53)	**0.009**
DAQS levels						
≥3	Ref		Ref		Ref	
<3	1.58 (1.33–1.87)	**<0.001**	1.30 (1.10–1.54)	**0.003**	1.18 (0.99–1.40)	0.060

The bold *P*-values represented statistically significant.

eGDR, estimated glucose disposal rate; DAQS, the dietary antioxidant quality score; OR, odds ratio; CI, confidence interval; Ref, reference.

Model 1: adjusted for age, gender, race, educational level, marital status, and PIR.

Model 2: adjusted for age, gender, race, educational level, marital status, PIR, smoking, dyslipidemia, CVD, CKD, overweight, decayed teeth, frequency of using dental floss, WBC, and anti-diabetic drug.

### Potential regulating effect of DAQS on association between eGDR and periodontitis

Table 3 showed the multiplicative interaction terms of eGDR and DAQS in association with periodontitis. The results suggested that there was a potential multiplicative effect between eGDR and DAQS on periodontitis (OR = 1.45, 95%CI: 1.05–2.00, *p* = 0.027). Associations between eGDR levels and periodontitis under different DAQS levels were analyzed (Table 4). After adjusting for covariates, an eGDR level of <8.296 was positively associated with increased odds of moderate to severe periodontitis, when individuals with a DAQS of <3 (OR = 1.67, 95%CI: 1.13–2.48, *p* = 0.012). Interestingly, this relationship was not significant among participants with DAQS of ≥3 (OR = 1.21, 95%CI: 1.00–1.46, *p* = 0.051).

**Table 3:** Multiplicative effect between eGDR and DAQS on periodontitis.

Variables	Unadjusted model		Model 1		Model 2	
	OR (95% CI)	*P*	OR (95% CI)	*P*	OR (95% CI)	*P*
eGDR	1.84 (1.61–2.09)	**<0.001**	1.33 (1.13–1.56)	**<0.001**	1.20 (1.00–1.44)	0.051
DAQS	1.33 (1.07–1.65)	**0.011**	1.08 (0.88–1.33)	0.455	0.99 (0.80–1.23)	0.925
eGDR * DAQS	1.39 (1.07–1.79)	**0.013**	1.46 (1.09–1.95)	**0.013**	1.45 (1.05–2.00)	**0.027**

The bold *P*-values represented statistically significant.

eGDR, estimated glucose disposal rate; DAQS, the dietary antioxidant quality score; OR, odds ratio; CI, confidence interval.

Model 1: adjusted for age, gender, race, educational level, marital status, and PIR.

Model 2: adjusted for age, gender, race, educational level, marital status, PIR, smoking, dyslipidemia, CVD, CKD, overweight, decayed teeth, frequency of using dental floss, WBC, and anti-diabetic drug.

**Table 4:** Association between eGDR and periodontitis under different DAQS levels.

Variables	Unadjusted model		Model 1		Model 2	
	OR (95% CI)	*P*	OR (95% CI)	*P*	OR (95% CI)	*P*
DAQS ≥3 (*n* = 7,518)						
eGDR ≥8.296	Ref		Ref		Ref	
eGDR <8.296	1.83 (1.61–2.08)	**<0.001**	1.33 (1.13–1.56)	**<0.001**	1.21 (1.00–1.46)	0.051
DAQS <3 (*n* = 2070)						
eGDR ≥8.296	Ref		Ref		Ref	
eGDR <8.296	2.55 (1.96–3.31)	**<0.001**	1.96 (1.40–2.75)	**<0.001**	1.67 (1.13–2.48)	**0.012**

The bold *P*-values represented statistically significant.

eGDR, estimated glucose disposal rate; DAQS, the dietary antioxidant quality score; OR, odds ratio; CI, confidence interval.

Model 1: adjusted for age, gender, race, educational level, marital status, and PIR.

Model 2: adjusted for age, gender, race, educational level, marital status, PIR, smoking, dyslipidemia, CVD, CKD, overweight, decayed teeth, frequency of using dental floss, WBC, and anti-diabetic drug.

Besides, this potential regulating effect of DAQS on association between eGDR and periodontitis was evaluated in different subgroups (Table 5). The significant multiplicative interaction terms of eGDR and DAQS were observed in age ≥60 years (OR = 3.86, 95%CI: 1.95–7.62, *p* < 0.001), female (OR = 1.68, 95%CI: 1.14–2.45, *p* = 0.009), BMI ≥ 25 kg/m^2^ (OR = 1.53, 95%CI: 1.01–2.33, *p* = 0.045), and non-DM (OR = 1.52, 95%CI: 1.06–2.18, *p* = 0.023) subgroups. Among these four subgroups, the association between eGDR and periodontitis was significant when DAQS <3 (all *p* < 0.05), while not significant when DAQS ≥3 (all *p* > 0.05) (Supplementary Table S4).

**Table 5:** Potential regulating effect of DAQS on association between eGDR and periodontitis in subgroups.

Variables	Multivariate model			
	OR (95% CI)	*P*	OR (95% CI)	*P*
Age subgroup	<60 (*n* = 6,540)		≥60 (*n* = 3,048)	
eGDR	1.36 (1.06–1.75)	**0.017**	0.70 (0.47–1.04)	0.075
DAQS	1.07 (0.86–1.33)	0.525	0.48 (0.25–0.93)	**0.031**
eGDR * DAQS	1.15 (0.78–1.68)	0.481	3.86 (1.95–7.62)	**<0.001**
Gender subgroup	Male (*n* = 4,755)		Female (*n* = 4,833)	
eGDR	1.36 (1.03–1.79)	**0.031**	1.08 (0.85–1.37)	0.511
DAQS	1.32 (0.95–1.84)	0.097	0.83 (0.63–1.09)	0.179
eGDR * DAQS	1.14 (0.74–1.78)	0.540	1.68 (1.14–2.45)	**0.009**
Overweight subgroup	BMI < 25 kg/m^2^ (*n* = 2,517)		BMI ≥ 25 kg/m^2^ (*n* = 7,071)	
eGDR	1.51 (1.04–2.20)	**0.030**	1.17 (0.94–1.46)	0.157
DAQS	0.97 (0.71–1.32)	0.855	1.00 (0.76–1.31)	0.992
eGDR * DAQS	0.82 (0.46–1.45)	0.486	1.53 (1.01–2.33)	**0.045**
DM subgroup	No (*n* = 7,784)		Yes (*n* = 1804)	
eGDR	1.16 (0.94–1.43)	0.154	1.75 (0.96–3.19)	0.068
DAQS	0.98 (0.79–1.23)	0.891	1.39 (0.36–5.34)	0.626
eGDR * DAQS	1.52 (1.06–2.18)	**0.023**	0.88 (0.21–3.72)	0.855

The bold *P*-values represented statistically significant.

DAQS, the dietary antioxidant quality score; eGDR, estimated glucose disposal rate; OR, odds ratio; CI, confidence interval; BMI, body mass index; DM, diabetes mellitus.

Age subgroups: adjusted for gender, race, educational level, marital status, PIR, smoking, dyslipidemia, CVD, CKD, overweight, decayed teeth, frequency of using dental floss, WBC, and anti-diabetic drug.

Gender subgroups: adjusted for age, race, educational level, marital status, PIR, smoking, dyslipidemia, CVD, CKD, overweight, decayed teeth, frequency of using dental floss, WBC, and anti-diabetic drug.

Overweight subgroups: adjusted for age, gender, race, educational level, marital status, PIR, smoking, dyslipidemia, CVD, CKD, decayed teeth, frequency of using dental floss, WBC, and anti-diabetic drug.

DM subgroups: adjusted for age, gender, race, educational level, marital status, PIR, smoking, dyslipidemia, CVD, CKD, overweight, decayed teeth, frequency of using dental floss, WBC, and anti-diabetic drug.

### Comparison on identification values of different IR-related indexes on periodontitis

**Figure 2:**
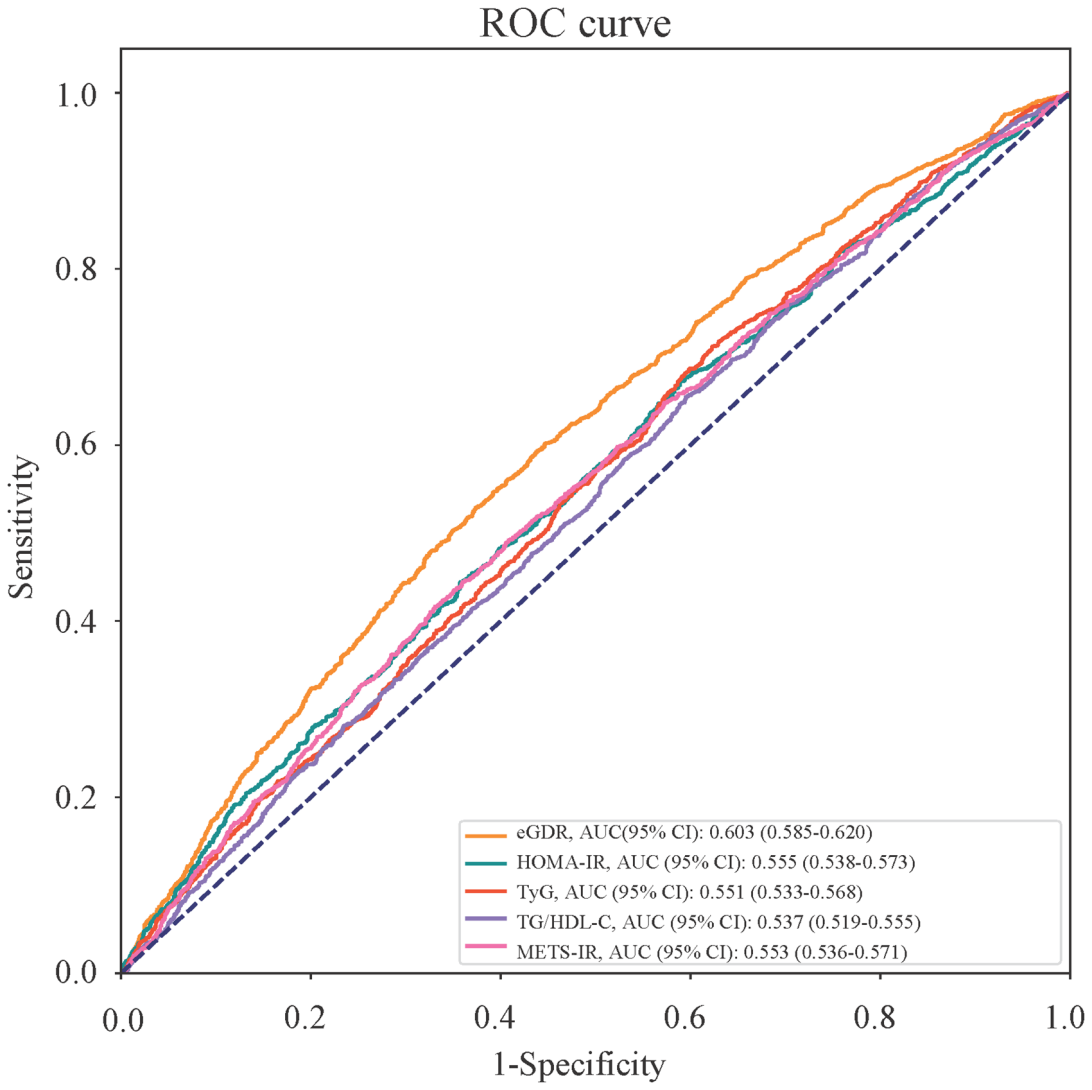
Receiver operating characteristic (ROC) curves of different IR-related indexes on identification of stage III/IV periodontitis.

Furthermore, we compared the values on periodontitis identification between eGDR and other IR-related indexes. Comparison of multiple IR-related indexes between Stage I/II periodontitis group and Stage III/IV periodontitis group was shown in the Supplementary Table S5. The average eGDR was significantly higher in stage I/II periodontitis group than that in stage III/IV periodontitis group (8.20 vs. 7.22), whereas the average levels of HOMA-IR, TyG, and METS-IR were significantly lower in stage III/IV periodontitis group than those in stage I/II periodontitis group (all *p* < 0.05). It could be clearly seen in the ROC curve (Figure 2) that eGDR has a relatively good value on periodontitis identification comparing to other IR-related indexes, with an AUC (95%CI) of 0.603 (0.585–0.620), and Delong test *p*-values of <0.001 (Table 6).

**Table 6:** Area under the curve (AUCs) and Delong test on different IR-related indexes for identification value in periodontitis.

Indexes	AUC (95% CI)	Delong test *Z*-values (eGDR vs. others)	Delong test *P*-values (eGDR vs. others)
eGDR	0.603 (0.585–0.620)		
HOMA-IR	0.555 (0.538–0.573)	5.56	**<0.001**
TyG	0.551 (0.533–0.568)	5.26	**<0.001**
TG/HDL-C	0.537 (0.519–0.555)	6.34	**<0.001**
METS-IR	0.553 (0.536–0.571)	5.39	**<0.001**

The bold *P*-values represented statistically significant.

AUC, area under the curve; IR, insulin resistance; CI, confidence interval; eGDR, estimated glucose disposal rate; HOMA-IR, homeostasis model assessment of insulin resistance; TyG, triglyceride-glucose; TG, triglyceride; HDL-C, high-density lipoprotein cholesterol; METS, the Metabolic Score for Insulin Resistance.

## Discussion

In the present research, we investigated the associations of eGDR and DAQS with periodontitis based on data from the NHANES database. The primary study results suggested that an eGDR level of <8.296 was associated with increased odds of moderate to severe periodontitis. A potential regulating effect of DAQS has been observed on relationship between eGDR and periodontitis, and this effect was also significant in age ≥60 years, female, BMI ≥ 25 kg/m^2^, and non-DM subgroups. Additionally, the value of eGDR in periodontitis identification was relatively better than other common IR-related indexes.

Epidemiological evidences suggested that the severity of periodontitis is higher in patients with DM than in healthy individuals. IR may play a crucial role in the pathogenesis of multiple diabetic complications and is reportedly induced in the gingiva of rodents with T2DM (11). In recent years, the bidirectional relationship between periodontitis and DM has been unveiled. Severity of periodontitis are increased and advanced in DM; and severe periodontitis in turn elicits adverse effects on DM through impairing insulin actions results from systemic microinflammation (30). Hence, looking for a simple and easily measured indicator to identify IR-associated periodontitis risk has important implications for reducing the current burden of disease in society. In clinical practice, the complex detection methods of insulin usually limit the evaluation of IR, and multiple indirect indexes have been proposed to reflect IR. Kalhan et al. (6) showed that the TyG index is associated with increased odds of moderate/severe periodontitis, especially in individuals with obesity, hypertension, and dyslipidemia. In Andriankaja et al.’s (31) study, they found that IR that assessed using the HOMA-IR index significantly predicts gingival/periodontal inflammation. Also, higher TG/HDL-C ratio, TyG, and METS-IR indices have been reported to be associated with a higher prevalence of periodontitis, and especially the METS-IR index, has more powerful predictive value than the other two (5). In the current study, we focused on the eGDR, which calculated using HbA1c, waist circumference, and hypertension, and has been shown to be strongly associated with various diabetic complications. The results suggested that a lower eGDR level was associated with increased odds of periodontitis, and its value in periodontitis identification was relatively higher than other four indexes, including HOMA-IR, TyG, TG/HDL-C and METS-IR. This indicated a prospect of eGDR in risk management of IR-related periodontitis.

Various mechanisms have been presented for explanation on the relationship between DM and periodontitis, including the following aspects. Excess glucose in the cell may have glucotoxicity or cause cellular damage, leading to intracellular generation of reactive oxidative species (ROS), which contribute to excess inflammatory cytokines production and attenuation in migration of innate immune cells and their bacteria-killing or clearance capacities (32, 33). Hyperglycemia-induced dysfunction of immune cells or glycated protein could cause OS, and chronic inflammation is induced by sustained infiltration of proinflammatory immune cells into periodontal tissue (34). Besides, diabetes-related change in oral microbiome, hyperlipidemia, obesity-related systemic chronic inflammation are common risk factors in periodontitis linked to DM (30). On the other hand, existing evidence has supported that dietary antioxidants may play important roles in periodontitis. Javid et al. (35) considered that daily consumption of antioxidant supplement may be beneficial in reducing serum levels of interleukin 6 in patients with T2DM combined with periodontal disease. A systematic review conducted by Tada et al. showed the VC intake and blood levels are negatively related to periodontitis, and the patients with a lower dietary intake or lower blood level of VC showed a greater progression of periodontitis than the controls (36). Recently, a cross-sectional study based on the NHANES database showed that a high quality of DAQS is related to the decreased risk of periodontitis, and this relationship is significant in participants without DM (37). According to the existing evidences on possible OS pathways under association between eGDR and periodontitis, we discussed the potential regulating effect of DAQS on this relationship. Our findings indicated that a higher level of DAQS may antagonize the increased risk of moderate to severe periodontitis that associated with lower eGDR levels. The DAQS consists of six micronutrients including VA, VC, VE, Se, Mg and Zn, and a higher score means a higher the level of dietary antioxidants. Comparing to previous studies that only focused on the relationship of single antioxidants and periodontitis, we investigated the effect of DAQS in eGDR and periodontitis, due to foods that people eat often contain complex antioxidants, not just a single antioxidant.

Additionally, this potential regulating effect was significant in age ≥60 years, female, BMI ≥ 25 kg/m^2^, and non-DM subgroups. In the United States, the prevalence of periodontitis is higher in the elderly population, and with age, metabolism-related chronic diseases and the decline of the body’s antioxidant function may affect the severity of periodontitis (38). Herein, this population may more sensitive to the regulating effect of a higher DAQS on periodontitis risk linked to lower eGDR. BMI and waist circumference were significantly associated with periodontitis, respectively, and there was an interaction effect between age and BMI (39). In addition, the possible reasons for the effect observed in non-DM instead of the DM population were that patients with DM have higher levels of inflammation, the role of nutrients is limited; and some hypoglycemic drugs may also affect the relationship between antioxidants and periodontitis. Nevertheless, these findings still need to be confirmed in further studies.

This was the first to explore associations of eGDR and DAQS with moderate to severe periodontitis based on the NHANES database, which has a good representation of the United States population. The value of eGDR for identifying stage III/IV periodontitis was confirmed, and the moderating effect of DAQS on IR-associated periodontitis risk was investigated. Diagnosis of periodontitis was through an examination of several periodontal sites by a professional dentist. Clinicians can use these findings to identify patients at high risk of periodontitis who have insulin resistance and low antioxidant nutrient levels, and prevent or slow the progression of strengths and limitations in our research. As a cross-sectional study, we could not periodontitis by enhancing patients’ antioxidant nutrient levels. However, there are strengths and limitations in our research. As a cross-sectional study, we could not clarify the causal relationships between eGDR and risk of periodontitis occurrence. In the NHANES, data were collected using retrospective questionnaires that may cause recalling bias. In addition, due to the limitation of the NHANES database, information on detailed dental treatment, such as subgingival scaling, diagnostic grading examinations and biochemical analyses, which could influence the analysis as potential confounding factors. In future studies, we plan to expand the sample scope by recruiting participants from various regions across multiple countries and conduct longitudinal research to enhance the generalizability of the findings. Additionally, stress may indeed influence the occurrence and development of periodontitis and should be included in future research as well.

## Conclusion

This study observed that a lower eGDR level is associated with increased odds of the moderate/severe periodontitis, and it has a value of periodontitis identification. A potential regulating effect of DAQS was found on relationship between eGDR and periodontitis, which was also significant in age ≥60 years, female, BMI ≥ 25 kg/m^2^, and non-DM populations. In clinical, focusing on DAQS in individuals with eGDR may help identify the potential risk of periodontitis development.

## Data Availability

Publicly available datasets were analyzed in this study. This data can be found at: the NHANES webpage: https://wwwn.cdc.gov/Nchs/Nhanes/.

## Footnotes

^1^ https://ods.od.nih.gov/HealthInformation/nutrientrecommendations.aspx
