# Supplementary material for: Regulating effect of dietary antioxidant quality on association between estimated glucose disposal rate and moderate to severe periodontitis
Source: Front Nutr. 2025 May 30;12:1561497. doi: 10.3389/fnut.2025.1561497 (PMC12162336; doi:10.3389/fnut.2025.1561497)
Supplement: Supplementary file 1 [file Table_1.docx]

Table S1. Variables with missing values

| Variables | N (%) |
| --- | --- |
| Educational level | 10 (0.1%) |
| Marital status | 5 (0.05%) |
| PIR | 757 (7.9%) |
| Smoking | 3 (0.03%) |
| Drinking | 463 (4.83%) |
| BMI | 16 (0.17%) |
| Physical activity | 2346 (24.47%) |
| CKD | 148 (1.54%) |
| Frequency of using dental floss | 79 (0.82%) |
| WBC | 14 (0.15%) |

PIR: poverty-to-income ratio, BMI: body mass index, CKD: chronic kidney disease, WBC: white blood cell.

Table S2. Characteristics of participants before and after multiple interpolation of missing data

| Variables | Before interpolation | After interpolation | Statistics | *P* |
| --- | --- | --- | --- | --- |
| Educational level, n (%) |  |  | χ^2^=0.524 | 0.770 |
| Below high school | 2179 (14.93) | 2182 (14.94) |  |  |
| High school | 2072 (20.71) | 2074 (20.71) |  |  |
| College and above | 5327 (64.35) | 5332 (64.36) |  |  |
| Marital status, n (%) |  |  | χ^2^=1.450 | 0.484 |
| Married | 5635 (62.84) | 5638 (62.84) |  |  |
| Never married | 1091 (10.57) | 1092 (10.58) |  |  |
| Others | 2857 (26.59) | 2858 (26.59) |  |  |
| PIR, n (%) |  |  | χ^2^=5.497 | 0.064 |
| ≤1.3 | 2592 (18.88) | 2838 (19.12) |  |  |
| 1.3-3.5 | 3153 (33.84) | 3450 (34.02) |  |  |
| >3.5 | 3086 (47.28) | 3300 (46.86) |  |  |
| Smoking, n (%) |  |  | χ^2^=1.275 | 0.259 |
| No | 5359 (55.64) | 5360 (55.63) |  |  |
| Yes | 4226 (44.36) | 4228 (44.37) |  |  |
| Drinking, n (%) |  |  | χ^2^=1.605 | 0.205 |
| No | 2376 (20.24) | 2532 (20.38) |  |  |
| Yes | 6749 (79.76) | 7056 (79.62) |  |  |
| BMI, kg/m^2^, Mean (S.E) | 29.11 (0.13) | 29.11 (0.13) | t=-1.43 | 0.159 |
| CKD, n (%) |  |  | χ^2^=0.029 | 0.865 |
| No | 8232 (89.76) | 8359 (89.76) |  |  |
| Yes | 1208 (10.24) | 1229 (10.24) |  |  |
| Frequency of using dental floss, times/week n (%) |  |  | χ^2^=0.886 | 0.347 |
| <3 | 4514 (44.98) | 4561 (45.01) |  |  |
| ≥3 | 4995 (55.02) | 5027 (54.99) |  |  |
| WBC, 1000 cells/uL, Mean (S.E) | 7.12 (0.04) | 7.12 (0.04) | t=-1.28 | 0.205 |

χ^2^: chi-square test, t: t test.

PIR: poverty-to-income ratio, BMI: body mass index, SE: standard error, CKD: chronic kidney disease, WBC: white blood cell.

Table S3. Covariates associated with periodontitis

| Variables | OR (95% CI) | *P* |
| --- | --- | --- |
| Age |  |  |
| <60 | Ref |  |
| ≥60 | 2.44 (2.02-2.93) | **<0.001** |
| Gender |  |  |
| Male | Ref |  |
| Female | 0.55 (0.49-0.62) | **<0.001** |
| Race |  |  |
| Non-Hispanic White | Ref |  |
| Non-Hispanic Black | 2.15 (1.73-2.68) | **<0.001** |
| Others | 1.66 (1.39-1.99) | **<0.001** |
| Educational level |  |  |
| Below high school | Ref |  |
| High school | 0.60 (0.48-0.75) | **<0.001** |
| College and above | 0.32 (0.26-0.39) | **<0.001** |
| Marital status |  |  |
| Married | Ref |  |
| Never married | 1.32 (1.10-1.58) | **0.004** |
| Others | 1.80 (1.55-2.10) | **<0.001** |
| PIR |  |  |
| ≤1.3 | Ref |  |
| 1.3-3.5 | 0.65 (0.54-0.77) | **<0.001** |
| >3.5 | 0.34 (0.29-0.41) | **<0.001** |
| Smoking |  |  |
| No | Ref |  |
| Yes | 1.86 (1.61-2.13) | **<0.001** |
| Drinking |  |  |
| No | Ref |  |
| Yes | 0.88 (0.75-1.03) | 0.107 |
| Physical activity |  |  |
| <450 | Ref |  |
| ≥450 | 0.90 (0.73-1.10) | 0.281 |
| Unknown | 1.28 (0.99-1.67) | 0.063 |
| Dyslipidemia |  |  |
| No | Ref |  |
| Yes | 1.39 (1.19-1.62) | **<0.001** |
| CVD |  |  |
| No | Ref |  |
| Yes | 2.49 (2.05-3.03) | **<0.001** |
| CKD |  |  |
| No | Ref |  |
| Yes | 2.11 (1.74-2.55) | **<0.001** |
| Overweight |  |  |
| No | Ref |  |
| Yes | 1.29 (1.13-1.48) | **<0.001** |
| Decayed teeth |  |  |
| No | Ref |  |
| Yes | 3.53 (2.98-4.20) | **<0.001** |
| Dental implants |  |  |
| No | Ref |  |
| Yes | 0.97 (0.65-1.45) | 0.874 |
| Frequency of using dental floss |  |  |
| <3 | Ref |  |
| ≥3 | 0.80 (0.71-0.91) | **<0.001** |
| WBC | 1.09 (1.06-1.12) | **<0.001** |
| Total energy intake | 1.00 (1.00-1.00) | 0.318 |
| Anti-diabetic drug |  |  |
| No | Ref |  |
| Yes | 1.87 (1.45-2.42) | **<0.001** |

The bold P values represented statistically significant.

OR: odds ratio, CI: confidence interval, Ref: reference, PIR: poverty-to-income ratio, DM: diabetes mellitus, CVD: cardiovascular disease, CKD: chronic kidney disease, BMI: body mass index, WBC: white blood cell, DAQS: the dietary antioxidant quality score, eGDR: estimated glucose disposal rate.

Table S4. Association between eGDR and periodontitis under different DAQS levels in subgroups

| Subgroups | DAQS ≥3 | | DAQS <3 | |
| --- | --- | --- | --- | --- |
|  | OR (95% CI) | *P* | OR (95% CI) | *P* |
| Age ≥60 | 0.70 (0.47-1.05) | 0.082 | 2.54 (1.16-5.56) | **0.021** |
| Female | 1.07 (0.83-1.37) | 0.609 | 1.98 (1.20-3.27) | **0.008** |
| BMI ≥25kg/m^2^ | 1.16 (0.93-1.45) | 0.182 | 1.82 (1.18-2.81) | **0.008** |
| Non-DM | 1.18 (0.95-1.45) | 0.127 | 1.65 (1.06-2.56) | **0.027** |

The bold P values represented statistically significant.

eGDR: estimated glucose disposal rate, DAQS: the dietary antioxidant quality score, OR: odds ratio, CI: confidence interval, BMI: body mass index, DM: diabetes mellitus.

Age≥60 subgroup: adjusted for gender, race, educational level, marital status, PIR, smoking, dyslipidemia, CVD, CKD, overweight, decayed teeth, frequency of using dental floss, WBC, and anti-diabetic drug;

Female subgroup: adjusted for age, race, educational level, marital status, PIR, smoking, dyslipidemia, CVD, CKD, overweight, decayed teeth, frequency of using dental floss, WBC, and anti-diabetic drug;

Overweight subgroup: adjusted for age, gender, race, educational level, marital status, PIR, smoking, dyslipidemia, CVD, CKD, decayed teeth, frequency of using dental floss, WBC, and anti-diabetic drug;

Non-DM subgroup: adjusted for age, gender, race, educational level, marital status, PIR, smoking, dyslipidemia, CVD, CKD, overweight, decayed teeth, frequency of using dental floss, WBC, and anti-diabetic drug.

Table S5. Comparation of multiple IR-related indexes between different periodontitis groups

| Variables | Total (n=4524) | Stage I/II periodontitis (n=1526) | Stage III/IV periodontitis (n=2998) | Statistics | *P* |
| --- | --- | --- | --- | --- | --- |
| eGDR, Mean (S.E) | 7.62 (0.06) | 8.20 (0.09) | 7.22 (0.07) | t=8.80 | **<0.001** |
| HOMA-IR, Mean (S.E) | 3.56 (0.11) | 3.05 (0.10) | 3.92 (0.15) | t=-5.07 | **<0.001** |
| TyG, Mean (S.E) | 8.64 (0.02) | 8.57 (0.02) | 8.68 (0.02) | t=-3.49 | **0.001** |
| TG/HDL-C, Mean (S.E) | 2.96 (0.07) | 2.87 (0.11) | 3.01 (0.09) | t=-1.06 | 0.296 |
| METS-IR, Mean (S.E) | 2.34 (0.01) | 2.31 (0.01) | 2.36 (0.01) | t=-4.73 | **<0.001** |

The bold P values represented statistically significant.

t: t test.

IR: insulin resistance, eGDR: estimated glucose disposal rate, SE: standard error, HOMA-IR: Homeostasis Model Assessment of Insulin Resistance, TyG: triglyceride-glucose, TG: triglyceride, HDL-C: high-density lipoprotein cholesterol, METS: the Metabolic Score for Insulin Resistance.
